# Food Waste Behaviour and Awareness of Malaysian

**DOI:** 10.1155/2022/6729248

**Published:** 2022-08-29

**Authors:** Chooi Lin Phooi, Elisa Azura Azman, Roslan Ismail, Jasmin Arif Shah, Evelyn Shin Rou Koay

**Affiliations:** ^1^Department of Crop Science, Faculty of Agriculture, Universiti Putra Malaysia, 43400 UPM, Serdang, Selangor, Malaysia; ^2^Department of Land Management, Faculty of Agriculture, Universiti Putra Malaysia, 43400 UPM, Serdang, Selangor, Malaysia; ^3^Institut Tanah Dan Ukur Negara (INSTUN), Kementerian Tenaga Dan Sumber Asli, Behrang, Tanjong Malim 35950, Perak Darul Ridzuan, Malaysia; ^4^Department of Agriculture Technology, Faculty of Agriculture, Universiti Putra Malaysia, Serdang 43400, Selangor Darul Ehsan, Malaysia; ^5^Faculty of Sustainable Agriculture, Universiti Malaysia Sabah, Sandakan 90000, Sabah, Malaysia

## Abstract

Food waste is a vast issue global, including in Malaysia. Food waste brings negative impacts, including increasing food production costs, impact on human health, and environmental degradation. Malaysian's animal- and plant-based diet preferences affected the desired food waste decomposition method as most of the methods only allow plant-based material to be utilized as food waste compost. The objectives of this study were to understand Malaysians' awareness of food waste behaviour and the food waste component for the decomposition. Malaysians usually produce more plant-based food waste than animal-based food waste. Most Malaysians have a high awareness of causes and impact of food waste, but they lack action on food waste reduction. Bio-compost is believed to be the most effective method to manage food waste, and most of them were willing to have it at home. However, some of them are unwilling to have a compost pile at home because there is no time to take care of it.

## 1. Introduction

Food waste is deemed against Sustainable Development Goal 12.3 (global food loss and waste). There was approximately 13.8% of food loss in the supply chain, such as harvest, transport, storage, and processing, in 2016 [1]. Industrialized countries are the major contributors to household food waste. Food waste is directly associated with social (e.g., health, equality), economic (e.g., increasing costs, consumption, resource efficiency, price volatility, waste management, commodity markets), and environmental (e.g., water, climate change, energy, depletion of resources, disruption of biogenic cycles due to intensive agricultural activities) impacts [2]. Resource-intensive food production causes damage to the environment; for instance, water and air pollution, deforestation, soil erosion, and greenhouse gas emissions occur during food production, storage, conveyance, and waste management [3]. Moreover, a lot of money could be saved by halting food waste.

Food waste is referred to as the food that is removed from the food supply chain during the phases of pre- and postconsumer. Food loss and waste are gradually decreased along the food supply chain during food quality inspection. Food loss particularly occurs from harvesting to retailers, food service providers, and consumers, whereas food waste usually occurs at the retail and consumer levels [4]. Food losses generally happen in the early phases of the food supply chain, while food waste occurs later. Food waste is viewed as a preventable food loss such as spoilage due to the mismanagement of temperature [5]. It may become an obstacle to achieving other goals, for example, reducing greenhouse gas emissions, improving food security and nutrition, lowering pressure on water and land resources, and increasing productivity and economic growth [4].

Reducing food waste can be measured using 3R—reducing, reusing, and recycling [6]. It is also related to our food-related routine and practices. The flow can be started from planning, shopping, storing, cooking, eating, and management [7]. Good planning strategies such as creating shopping lists, meal plan combinations, and inventory checking before shopping showed low food waste reported [7], and trying not to fall into the commercial trap such as “Buy one get one” while shopping. Purchase should be based on the need for the food on specific days. Traditional practice was used for waste management when there were fewer techniques and knowledge during ancient times. Food waste can be reduced by preparing and consuming food in a sufficient amount. The leftovers can be recycled to produce commercial products including ethanol and biofuel. Food waste behaviour can be minimized by planning purchases, cooking, and eating practices [6, 7].

Sustainable Development Goal 12 ensures sustainable consumption and production patterns targeted (12.3) halve per capita global food waste at the retail and consumer levels and reduce food losses along the production and supply chains, including postharvest losses and (12.5) substantially reduce waste generation through prevention, reduction, recycling, and reuse by 2030 [1]. Food waste causes vary according to countries [8]; however, food waste behaviours and composition in Malaysia remain unclear. Therefore, the food production chain and food waste management can be adjusted with consumer behaviour and awareness.

The study questions were as follows: (1) Do Malaysians know the impact of food waste? (2) What are their actions related to food waste? (3) Do their eating diet affect the food waste composition? and (4) Does Malaysia provide a food waste management system? Hence, the objectives of this study were to understand the food waste behaviour and awareness of Malaysian and to understand the food waste component for the decomposition of Malaysia for better waste management.

## 2. Study Approach

**Figure 1:**
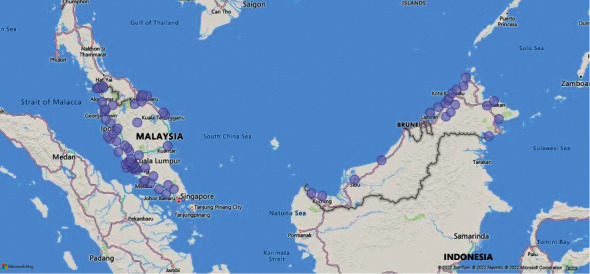
Distribution of the respondents in Malaysia.

This study conducted an online survey with two demographic sections and food waste-related questions. Food waste is determined by physiological factors and food-related routines [9]. Gender and marital status are closely related to food waste behaviour, where females and married are prone to reduce food waste [10]. Thus, the distribution of the respondents was included in the survey (Figure 1). High moral attitudes brought low food waste behaviour [10].

The 3R practices are comprehensive and holistic measurements. Anticipated guilt (emotional) and a sense of community (social) were the drivers of food waste behaviour and are positively related to the practicing of 3R [6]. Thus, the sections on eating behaviour, food preparation behaviour, plate waste behaviour, and cognition were to understand respondents' behaviour better. Furthermore, high awareness of food waste impact and environmental knowledge showed high 3R practices [6]. Therefore, the section on the awareness of food waste impact and its management was generated. Calculation of the amount of food waste in grams was presumed, and the respondents were guided to assume one spoon equal to 50 grams [11].

The survey is performed based on a probability sample of 400 Malaysian respondents (Table 1; Figure 1) to represent the population of Malaysia (32, 814, 249). The survey questions were multilingual, including English, Malay, Simplified Chinese, and Traditional Chinese, and were modified and adapted according to the study [9, 11, 12]. The survey was initiated in November 2021 and ended in January 2022. The respondents were approached through social media such as e-mail, WhatsApp, Telegram, Instagram, and Facebook.

## 3. Results and Discussion

### 3.1. Causes of Food Waste

**Figure 2:**
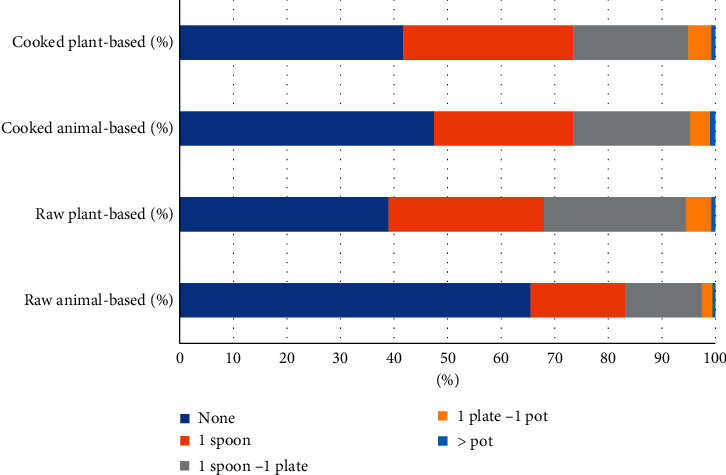
Food waste composition such as raw animal-based, raw plant-based, cooked animal-based, and cooked plant-based wasted as none, one spoon, one spoon to 1 plate, one plate to 1 pot, and more than one pot. The assumption was made as one serving spoon is equal to 50 grams, one plate is equal to 500 grams, and one pot is equal to 1000 grams.

**Figure 3:**
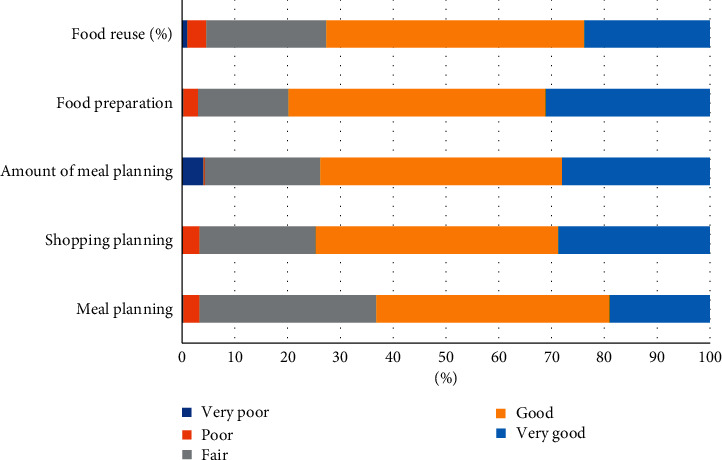
Household skills claimed by respondents.

The majority (95.75%) of the respondents were plant and animal consumers (Table 1). The food waste composition such as raw animal-based, raw plant-based, cooked animal-based, and cooked plant-based was more than 50% (Figure 2). About half of the respondents (47.50%) claimed no cooked animal-based waste (Figure 2). Only 1% of the respondents declared that they discarded more than one pot of food. However, most of them claimed that they were good at food planning (Figure 3), and this may probably be due to the consumers having different tastes in their cooking. It was found that their cooking can modify their cooking manner to suit consumers' tastes. On the flip side, the consumers should be tolerant of the taste of the food. Therefore, consumer preference should be observed and asked before cooking. In addition, food waste management on animal-related foods is required since most of the composting methods are not suitable for it.

More than half of the respondents (61%) stated that raw plant-based food was discarded daily (Figure 2). Most of them mentioned that there were only 26% of raw plant-based food discarded per day. This indicated that Malaysians have good behaviour in practices of reducing food waste. Some of the raw plant-based foods are anyhow avoidable. Nearly half of avoidable food (46%) such as fresh, raw, or minimally processed state was wasted in the United Kingdom (UK) [13].

Less than half of Malaysians claimed that they do not waste any cooked plant- and animal-based food, with 41.75% and 45.70%, respectively. According to the results, most Malaysians tend to waste cooked food. Hence, parents should start home education for their children, and parents should become their role models to know the preciousness of the food. Religions may also play an important role in reducing food waste. For formal education, teachers can educate the students on how to grow vegetables to know the preciousness of the food from a young age. Approximately 27% of cooked or prepared food is wasted, and 20% is ready to consume when purchased [13]. In addition, starchy foods are the most commonly wasted food after being prepared [13].

**Figure 4:**
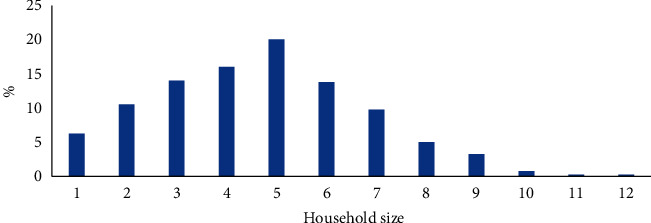
Household size of the Malaysians.

Most of the Malaysians have 5 (20.05%), 4 (16.04%), and 3 (14.03%) household size (Figure 4). Food waste amount was influenced by sociodemographic variables such as education, employment, income, and the number of members in the household. Countryside households donated less to food waste generation than town areas [14]. Behaviours such as buying the best offers and eating out frequently increased food wastage [14].

The nutritional benefits of meals such as school lunches and home meals are reduced by plate waste [15]. The preparation method highly affected the acceptance rate of plate waste [15]. For instance, well-prepared and easy-to-consume foods such as mashed potatoes and heated fries had high acceptance, but mashed potatoes were wasted less [15]. Uncut apples had lower acceptance (23%) and greater waste (62%) compared with applesauce (37% acceptance, 23% waste) [15].

In Danish households, the ratio of unavoidable food waste to avoidable food waste was 2 to 3 (based on kg per household per year) [16]. In Finnish households, food waste including vegetables, home-cooked food, and milk products ranged from 0 to 160 kg/year [17]. Singles tend to waste more food than others [16, 17]. In Mamelodi, 58% of households in developing countries wasted the largest portion of porridge, while 26% and 16% of households mainly wasted rice and bread, respectively [18].

**Figure 5:**
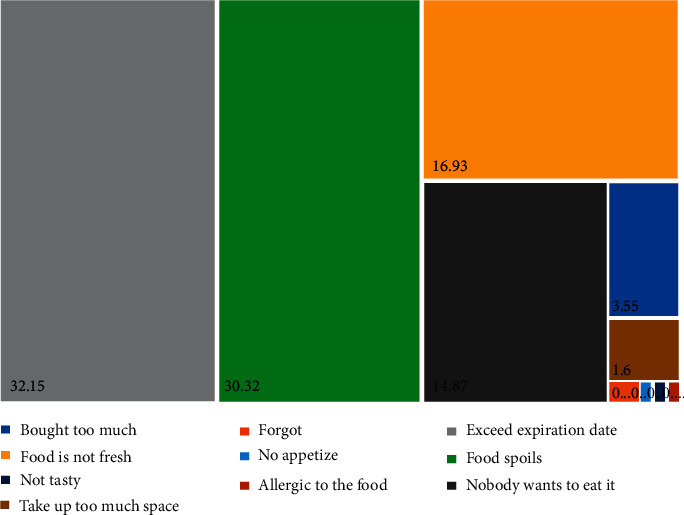
Reasons for food waste declared by respondents.

Malaysians declared that the reasons for food waste are exceeding the expiry date (32.15%), followed by food spoils (30.32%) and food not being fresh (16.93%) (Figure 5). Some of them also declared that they forget about the food (0.25%). In-home consumer waste is affected by poor purchase and meal planning, excess buying (influenced by over-large portioning and package sizes), confusion over labels (e.g., best before and use by), and poor in-home storage [4]. Knowledge about “best before” and “used by date” was lacking among the consumers, and they tend to be misled by the consumers to throw away the edible food. Therefore, educating them to know the difference in the expiry date is very important for reducing food waste. Perhaps, Malaysians may also overestimate their household skills, especially in meal planning, for the quantity of food they need (45.8%) (Figure 3). Thus, Malaysians paid less attention to food waste due to their personal behaviour.

The primary triggers for food waste were (1) the preparation of food, including porridge and rice [17, 18]; (2) spoilage of food such as bread reaching the expiry date before being consumed [17, 18]; (3) buying in excess [17, 18]; and plate leftovers [17]. Food such as vegetables, fruits, and berries are usually wasted when people do not consume them adequately [17]. Out-grading or quality control, damaged or inadequately prepared items, overstocking or over-preparation of food, unpurchased speciality holiday food, damaged packaging, and routine kitchen preparation waste lead to food waste at the retail and institutional levels [19].

**Figure 6:**
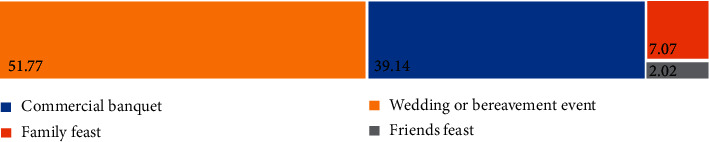
The most serious food waste banquet believed by Malaysians.

**Figure 7:**
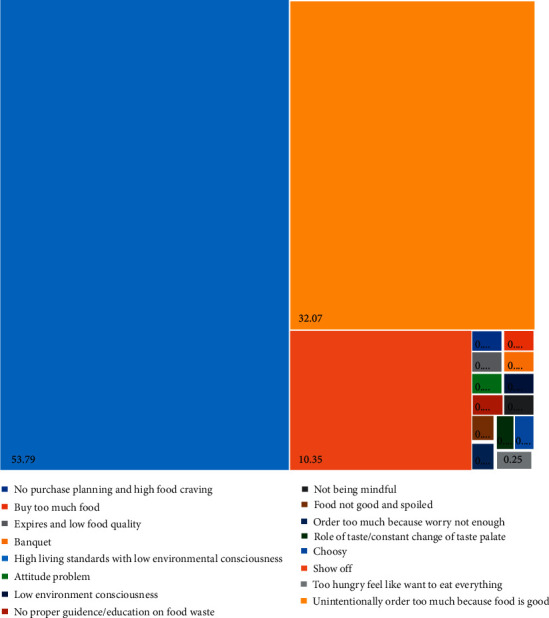
Current food waste reasons believed by Malaysians.

Malaysians believed the most serious food waste banquet is a wedding or bereavement event (51.77%), followed by a commercial banquet (39.14%), a family fest (7.07%), and a friend feast (2.02%) (Figure 6). A wedding or bereavement event is believed to be the event that leads to the most serious food waste. These conditions were similar to Macau [12], and this may be due to the reason of high living standards and low environmental conscience (Figure 7) in Macau [12]. The high living standard also increased the total food waste [20, 21]. Nonetheless, household income may not show a clear factor in food waste [22]. People are demanding exquisite food and thus encourage the people to supply more food during the event. People are not precious the food on hand compared to a few eras ago, which less developed living environment. Besides, Malaysians tend to order too much unintentionally, which is beyond their eating ability (Figure 7), and hence, they need to balance their greed and needs, especially during wedding or bereavement events. Moreover, people like to show off their status and trigger them to design a delicacies wedding or bereavement event menu. Yet, the guests are taking too much or not suitable for the guests to consume. The chefs may not be able to maintain cook the food to standard for the whole event.

### 3.2. Awareness of Food Waste Impact

**Figure 8:**
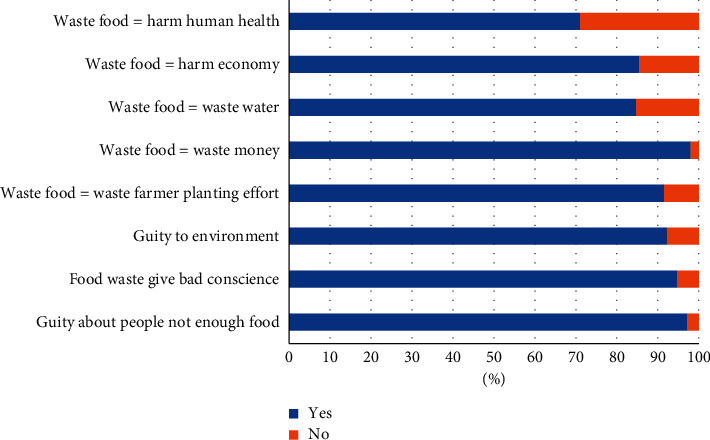
Perception of food waste.

**Figure 9:**
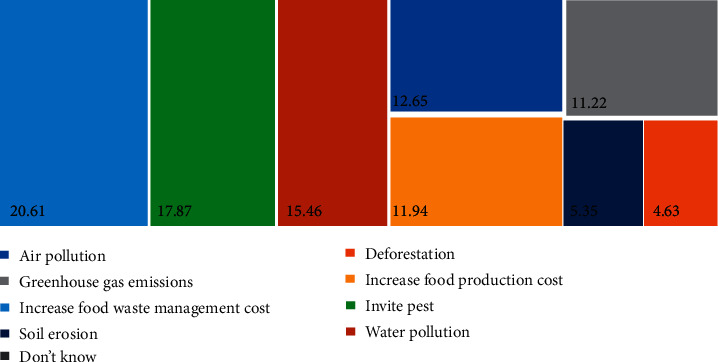
Perception of food waste impact.

Malaysians have a high awareness of food waste. More than 70% of Malaysians feel that wasting food is a guilty action, a waste of money, natural recourses, including water, and farmer planting efforts (Figure 8). Beliefs such as feeling guilty decreased food waste generation [14]. Most Malaysians also believe that food waste will increase the waste management cost (20.61%), followed by pest invitation (17.87%) and water pollution (15.46%) (Figure 8). It was a good sign when there was a reduction in food waste, as this indicated that they understood and knew about the impact of food waste. However, only 0.72% of Malaysians stated that they do not know about the impact of food waste (Figure 9).

Only 11.94% of Malaysians think food waste will increase food production costs (Figure 9). Food waste will waste money and cause harmfulness to the economy. The price transmission all along the supply chain is affected by an initiative to reduce food loss or waste. Nevertheless, the exact effect of food loss and waste reduction relies on how effectively the price changes are transmitted and how closely the markets are incorporated. Therefore, a major aspect is the distance or proximity to the location of the reduction [4]. For illustration, the local food security impact may strengthen by reducing losses on small farms in lower-income countries, and thus, surplus food will be available in the local area. However, reducing food waste in high-income consumers does not mean that surplus food is available for the poor and food-insecure people in a distant country, and their level of food insecurity remains the same. Food loss and waste reduction strategies are determined by the level of food insecurity in a country [4].

Food loss and waste imply poor resources use and adverse environmental impacts. It is estimated that more pressure will be put on natural resources as the rising incomes and growing population will increase food demand [4]. Reducing food loss and waste is crucial to improving the use of natural resources; however, it will contribute to lower greenhouse gases emissions per unit of food consumed directly as there will be more food reaching the consumer for a given level of resources used [2, 4]. Moreover, the growth of the economy also changed moral and ethical relations [23], increased food waste [20, 21], reduced environmental quality, and evaluated the carbon dioxide emission in Malaysia [24].

### 3.3. Awareness of Food Waste Management

**Figure 10:**
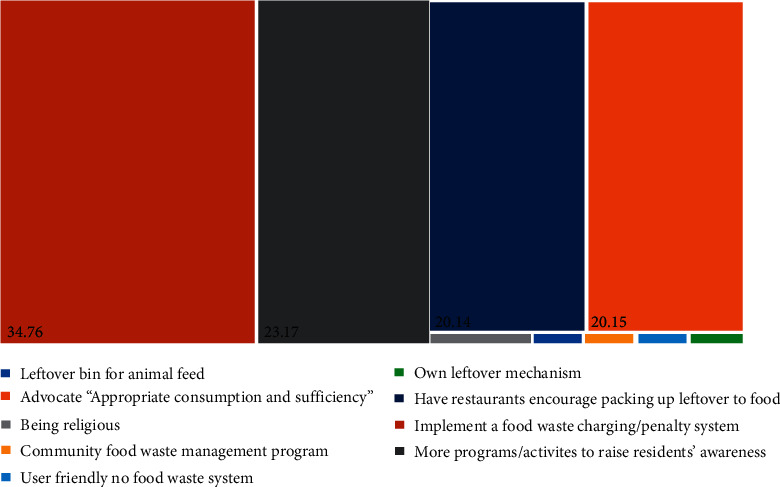
Effective ways to reduce food waste believed by respondents.

The effective way to reduce food waste believed by Malaysians are implementing a food waste charging or penalty system (34.76%), more programs or activities to raise residents' awareness (23.17%), and having restaurants encourage leftover packing (20.40%) (Figure 10). Most Malaysians believe that the penalty for food waste can be the solution to reduce food waste as most of them think food waste is equal to wasting money (Figure 8). However, this would be limited to certain restaurants, and Malaysian may not keep on with the eating habit that does not waste food at home. Besides that, the restaurant owner may not claim the penalty with their customers as this action may affect their business and profit. Arguments might occur regarding who will be the party to pay for the penalties of food waste between the collection of penalties to the upper (restaurant staff), middle (trash collector), and bottom (digestion and factory staff) stream of the food waste management.

**Figure 11:**
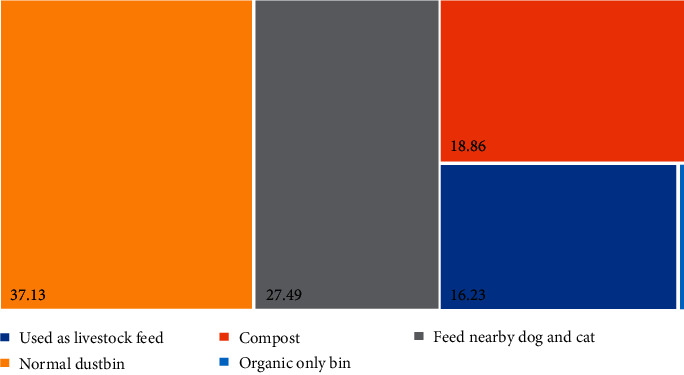
Food waste management claimed by respondents.

**Figure 12:**
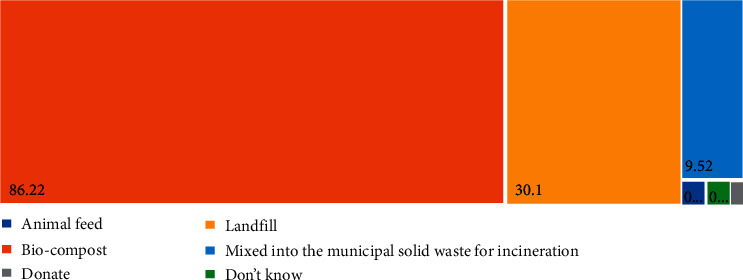
The effective ways for reducing food waste that Malaysians believe.

**Figure 13:**
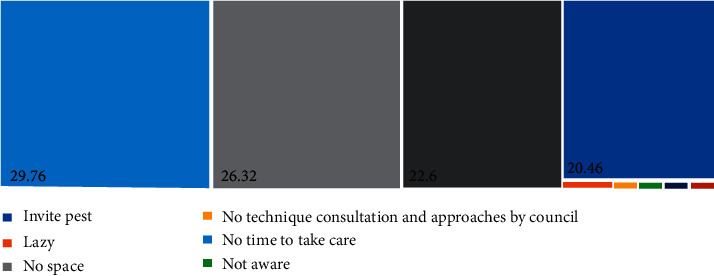
Willingness to have a composting pile at home.

Most Malaysians discard their food waste to the normal dustbin (37.13%), followed by feeding nearby dogs and cats (27.49%) and compost (18.86%) (Figure 11). Malaysians believed that the most effective way to manage the food is bio-compost (86.22%), followed by landfill (30.1%) and mixing into the Manipal solid waste for incineration (9.352%) (Figure 12). More than half of the respondents (67.00%) believed food waste dumpsite in Malaysia is inappropriate (Table 2). It might take several years if they relied on the government to provide an appropriate dump site for food waste. Therefore, being self-responsible for food waste is important for keeping a clean and sustainable environment. Fortunately, most Malaysians (75.75%) are willing to have a composting pile at home (Table 2). However, only 59.50% of Malaysians are willing to do more for food separation. The remaining were not willing to have a compost pile at home due to the reasons of no time to take care of it (29.76%), followed by no space (26.32%) and smelly (22.60%) (Figure 13).

This finding was similar to what the people who live in Macau believed. About 70% of people in Macau think that bio-compost is the best way to treat food waste [12]. However, they are not willing to have a compost pile at home due to the reasons of pest attraction (20%), smell (22%), no extra space allowed (25%), no time to take care of it (28%), and other reasons (4%) [12]. Approximately 70% of people in Macau are willing to pay more for food waste separation; nevertheless, it is correspondent to the level of income and age [12].

Malaysians do have environmental and food waste consciousness (Figures 8 and 9); however, they prefer to waste food first and manage the food waste against environmental impact. Prevention is better cure. Food waste prevention should educate young to avoid the consequences, which cost money, the environment, and physical and mental health. For instance, food waste management contributed up to 6% of food waste-related impact in Europe [25].

Despite food waste prevention, the food waste management approach is composting, anaerobic digestion, incineration, thermal conversion, landfilling in-sink food, drying for animal feed, co-digestion at waste water treatment plant (WWTP), and bio-valorisation, and yet, they do bring both positive and negative impacts on water and energy consumption and offsets [5]. Landfill food waste is also suggested to be banned as it is not eco-friendly [26]. Therefore, food waste management is costly in terms of not only money but also the environment.

Cooked food wastes are more suitable for transforming into feedstocks than raw ones [27]. This is because cooked food waste was high in nutrients than the raw ones; nonetheless, cooked food wastes had lower temporal variability [27]. Black soldier fly is the most economically favourable food waste treatment [28]. However, food waste can also be degraded by anaerobic digestion economically with the existence of an anaerobic digestion plant [28]. Black soldier flies food waste treatment reduced 20% of the biomethane potential [28].

A convenient, easy, and household-scale food waste management system can be introduced in Malaysia. This system was introduced to provide an easy way for Malaysians to manage food waste, and this also can encourage Malaysians to be responsible for their food waste. Food waste digestion has various aerobically (thermal composting, vermicomposting) and anaerobic methods (Bokashi).

Thermal compost experiences phases such as mesophilic, thermophilic, and stable phases. The thermophilic phase has a peak temperature range between 45 and 70°C [29]. Thermal composting and vermicomposting are time-consuming (more than 12 weeks) and not suitable for routine use [30, 31]. Besides, thermal composting is a precomposting for 9 days, followed by 2.5 months of vermicompost, which can produce safe compost with reduced mass, pathogen, and moisture management [32]. However, it was still considered a time-consuming method, and it required obtaining worms as the sources of vermicompost. After vermicomposting, the worm is separated from the compost manually or by a factory-scale separator [33]. It is not suitable for the household scale and is quite labour-intensive.

Bokashi would be a suitable choice at the household level to manage food waste. Bokashi is a process, meaning fermented organic matter using effective microorganisms (EM), molasses, and water. The main advantage of Bokashi is short processing time (7 to 21 days) [2, 34–36]. Furthermore, Bokashi only required a small corner or space to digest the food waste. Apart from that, Bokashi continuously allowed the top-up of food waste (raw materials) in a pile. It is suitable for household scale to carry out composting with a small amount of food waste, which is 1 spoon to 1 plate (Figure 2), and this can fulfil the Malaysians who are not willing to have a compost pile at home (Figure 13). Bokashi can be classified into aerobic and anaerobic digestion [35]. However, it was deemed that it is anaerobic at the early stage and aerobic at the later stage for greater product stability [35]. High ammonium and low nitrate concentrations were found at the Bokashi low oxygen level [35].

A relatively good food waste management method can be introduced to Malaysians through social media or stricter ways involving the law and regulations. Malaysian news media can introduce the method to manage food waste; thus, the elder can have the basic knowledge of it. Besides, the community of apartments can have a community compost pile for their community garden. The community can also strengthen their relationships by carrying out this action. In Taiwan, food waste is collected daily at a specific time in the evening in a separate bin. Therefore, Taiwan has no dumpsite with food waste that can be disrupted by stray cats and dogs.

## 4. Conclusions

More than 50% of Malaysian wasted food daily. Most Malaysians claimed they have good household skills but are low in action to manage them. Furthermore, the high living standard makes the community have low environmental consciousness even though they understand wasting food is equal to wasting money and increased food waste management cost. Therefore, they believed implementing of food waste charging/penalty system is the most effective way to reduce waste. They tend to discard food waste to normal dustbins even though they believed the best management is bio-composting. Malaysians understood the impact of food waste and the relatively good management method. However, there is not much action done to reduce food waste. Therefore, easy and convenient composting such as Bokashi can be introduced to Malaysians.

## Figures and Tables

**Table 1:** Respondent's characteristics.

Characteristics	Respondent (%)
Gender	
Female	67.50
Male	32.50

Marital status	
Single	50.25
In a relationship	7.50
Married	41.25
Divorced	1.00

Household income (RM)	
<4500 (B40)	44.50
4500–11000 (M40)	39.50
>11000 (T20)	11.00

Higher education	
Primary school	0
Secondary school	2.50
Diploma degree	10.25
Bachelor's degree	53.75
Master's degree	16.75
Doctorate degree	16.75

Occupation	
Government	34.25
Private	26.00
Freelancer	5.25
Student	32.50
Retired	0.75
Unemployed	1.25

Current eating diet	
Plant and animal eater	94.50
Pescatarian (plant and fish)	1.25
Flexitarian (part-time vegetarian)	2.75
Vegetarians (plant, dairy, and animal by-products)	1.25
Vegan (plant only)	0.25

**Table 2:** Perception of respondents on having an appropriate food waste dumpsite in the current location, having a compost pile at home, and paying more for food separation.

Perception	Yes (%)	No (%)
Have an appropriate food waste dumpsite in the current location	33.00	67.00
Willingness to have a compost pile at home	75.75	24.25
Willingness to pay more for food separation	59.50	40.50

## Data Availability

The figure data used to support the findings of this study have been deposited in the figshare repository (10.6084/m9.figshare.20436465).
